# Endovascular Revascularisation versus Open Surgery with Prosthetic Bypass for Femoro-Popliteal Lesions in Patients with Peripheral Arterial Disease

**DOI:** 10.3390/jcm12185978

**Published:** 2023-09-15

**Authors:** Gladiol Zenunaj, Pierfilippo Acciarri, Giulia Baldazzi, Alessio Mario Cosacco, Vincenzo Gasbarro, Luca Traina

**Affiliations:** 1 Unit of Vascular and Endovascular Surgery, University Hospital of Ferrara, 44124 Ferrara, Italy; filippo.acciarri@gmail.com (P.A.); gsv@unife.it (V.G.); trainaluca@yahoo.it (L.T.); 2 Department of Translational Medicine for Romagna, School of Vascular Surgery, University of Ferrara, 44121 Ferrara, Italy; giulia01.baldazzi@edu.unife.it (G.B.); alessio.mario.c@gmail.com (A.M.C.)

**Keywords:** endovascular procedures, femoral popliteal bypass, peripheral arterial disease, prosthetic graft

## Abstract

**Aim**: Complex atherosclerotic femoro-popliteal lesions have traditionally been treated with bypass surgery. A prosthetic graft is used to save the vein graft for more distal revascularisations or when a vein graft is unavailable. The endovascular approach has gained popularity and is offered as a first-line strategy for complex lesions. This study aimed to evaluate whether endovascular procedures can be used as a first-line treatment strategy for complex native femoro-popliteal lesions over open surgery with prosthetic bypass in patients with peripheral arterial disease (PAD). **Methods**: This single-centre retrospective observational study was conducted between 2013 and 2021; it included patients with symptomatic PAD who required limb revascularisation at the femoro-popliteal segment and who had complex lesions. The primary endpoints analysed were technical success, primary patency, freedom from clinically driven target lesion revascularisation (cdTLR), freedom from major adverse limb and cardiovascular events (MALE and MACE, respectively), freedom from limb loss, and survival. The secondary endpoints were length of in-hospital stay, and duration and costs of the procedure. **Results**: We identified 185 limbs among 174 suitable candidates for comparison, wherein 105 were treated with an endovascular procedure and 80 with a femoro-popliteal prosthetic bypass. Most patients in both groups presented with chronic limb-threatening ischaemia, and >90% of them had an American Society of Anesthesiologists (ASA) physical status classification of >3. The endovascular group had more octogenarians (*p* = 0.02) and patients with coronary disease (*p* = 0.004). The median follow-up was 30 months. The technical failure rate for endovascular procedures was 4.7%, versus 0% in the open group (*p* = 0.047). Freedom from MACE was similar in both groups. The endovascular group showed superior primary patency (*p* < 0.0001), cdTLR (*p* < 0.0001), MALE (*p* < 0.0001), and freedom from limb loss (*p* = 0.0018) at 24 and 48 months. Further analysis performed for the open above-the-knee sub-group showed that the aforementioned endpoints were similar between the groups at 12 months and were better in the endovascular group at 24 and 48 months. Procedural time and in-hospital stay were longer in the open group than in the endovascular group (*p* < 0.0001 and *p* < 0.001, respectively). The procedural cost in the endovascular group was 10-fold lower than that in the prosthetic bypass group. **Conclusions**: Endovascular procedures are safe for treating complex femoro-popliteal lesions in patients at a high risk for surgery and show better outcomes at 24 months than prosthetic bypasses do. The latter may be considered as an alternative should endovascular treatment fail.

## 1. Introduction

The incidence of critical limb-threatening ischaemia is increasing as the age of the population and prevalence of cardiovascular disease increase, leading to significant socio-economic impact [1]. For decades, an endovascular approach has been available for less complex lesions (TASC II A and B according to the Trans-Atlantic Inter-Society Consensus for the Management of Peripheral Arterial Disease) [2] and patients at a high risk for surgery [3,4]. In contrast, open surgery was offered to patients with more complex (TASC II C and D) lesions who were considered to be at a low or moderate risk for surgery [3,4]. Significant improvements in endovascular technology and improved knowledge of atherosclerotic lesions have overcome these recommendations. Currently, the endovascular approach is offered as a first-line strategy, even for complex lesions. However, open revascularisations performed using a vein graft have demonstrated greater efficacy and durability in comparison to the endovascular procedures over time [4,5]. Unfortunately, numerous situations exist where a suitable vein graft is unavailable, and many practitioners prefer prosthetic grafts. In cases where a femoro-popliteal bypass above the knee is required and a suitable vein is available, the tendency is to save the vein for more distal revascularisations [6,7]. In this study, we sought to evaluate whether using the endovascular approach for complex femoro-popliteal lesions could be more advantageous than using a prosthetic bypass in real-world practice for patients with peripheral arterial disease (PAD).

## 2. Materials and Methods

This was a single-centre retrospective observational study performed between January 2013 and December 2021, and included patients with symptomatic PAD treated by either open or endovascular revascularisation for native complex femoro-popliteal lesions (classified as TASC II C and D according to the Trans-Atlantic Inter-Society Consensus for the Management of Peripheral Arterial Disease) [2]. Clinical limb presentation was described using the Rutherford classification [8].

Patients requiring procedures in below-the-knee vessels other than femoro-popliteal revascularisation were excluded, apart from patients presenting with a no-option below-the-knee anatomy upon instrumental evaluations [1]. Additionally, patients with a history of either open or endovascular revascularisation at any level of the target lower limb arteries were excluded from this study to avoid influencing the outcomes of the first-line strategy [4,9].

The study was approved by the Institutional Review Board of the Independent Ethics Committee and was registered with N. ER.FE.2022.15. The requirement for patient consent was waived given the retrospective nature of the study. To enhance clarity, the study adhered to the STROBE guidelines [10]. Data regarding patient demographics, co-morbidities, clinical limb presentation, characteristics of the target vessel, and run-off quality before and after the procedure were collected and analysed. The characteristics of the interventions regarding vascular access, devices used, and their characteristics were also analysed.

Technical success was defined as evidence of successful revascularisation with a residual stenosis of <30% after angioplasty, and the absence of flow-limiting lesions. Major amputation was defined as any lower-extremity amputation through, or proximal to, the ankle joint. Minor amputation was defined as any lower-extremity amputation distal to the ankle joint.

The primary endpoints were primary patency, freedom from clinically driven target lesion revascularisation (cdTLR), freedom from major adverse limb events (MALE) (including major amputation and any intervention to restore patency), freedom from major adverse cardiovascular events (MACE), and freedom from all causes of death. The secondary endpoints were length of hospital stay, duration of the procedure, and cost of materials.

Regarding statistical analysis, continuous variables are presented as means ± standard deviations for normally distributed data; variables are presented as percentages. Standardised mean differences were used to compare the prevalence between groups, with statistical significance set at *p* < 0.05. Primary endpoints were analysed using Kaplan–Meier curves, and statistical significance was evaluated using the log-rank test.

## 3. Results

**Figure 1:**
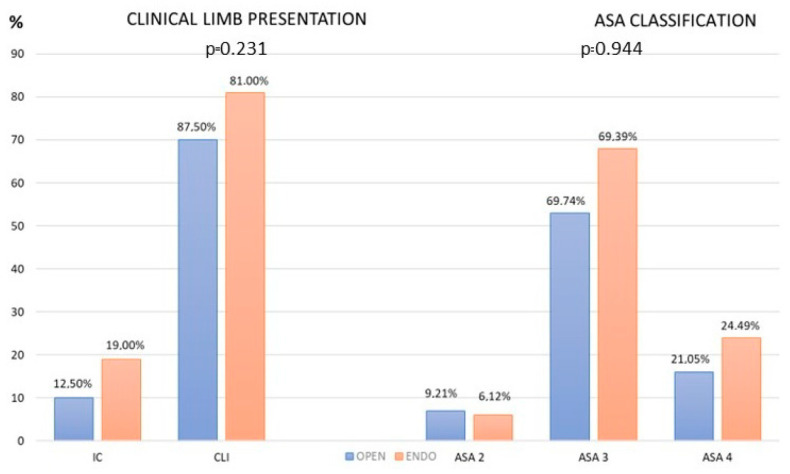
Clinical presentation (on the left) and distribution of the American Society of Anesthesiologists (ASA) Physical Status Classification System scores (on the right) of patients included in the open and endovascular groups. IC: intermittent claudication; CLI: critical limb ischemia.

Among 174 patients, we identified 185 limbs that were suitable for comparison: 105 of these limbs underwent treatment using an endovascular approach and 80 using a femoro-popliteal prosthetic bypass. Most patients presented with critical limb-threatening ischaemia with no significant difference between the two groups, *p* = 0.231 (Figure 1). In comparison to the endovascular group, the open group had fewer octogenarians (*p* = 0.02), and no nonagenarians. In the endovascular group, a significant prevalence of patients suffering from coronary disease was noted (*p* = 0.004) (Table 1). In both groups, most of the patients had an American Society of Anesthesiologists (ASA) physical status classification of 3 and 4, with no statistical significance observed between the groups, *p* = 0.944 (Figure 1).

In the open group, the instrumental evaluation consisted of a duplex ultrasonography (DUS) in all cases, a computed tomography (CT) scan in 69 cases, and in the remaining cases, a digital subtraction angiography (DSA). In the endovascular group, in all cases the instrumental evaluation consisted of a DUS assessment and a subtracted angiography. The median follow-up was 30 months (range: 12–48 months) and consisted of clinical and ultrasonographic examinations at 1 and 6 months, and annually thereafter.

Run-off vessel quality was worse in the endovascular group than in the open group, with 11 cases with no-option anatomy in the former versus none in the latter (Table 2). In these cases, achieving at least one runoff vessel was mandatory, and in all cases, it consisted of the peroneal artery. More limbs with three run-off vessels were observed in the open than in the endovascular group, with 28 vs. 10 limbs (*p* < 0.001), respectively.

Procedural characteristics. In the endovascular group, 52 procedures were performed percutaneously by puncturing the femoral artery under ultrasound guidance; haemostasis was achieved using a percutaneous closure device (Angio-Seal VIP, 6 Fr; Terumo Medical Corporation, Somerset, NJ, USA). Vascular access was achieved in the remaining cases using the femoral cut-down technique. Two different approaches were used in the endovascular group because of randomisation in another study, which evaluated the outcomes between the two vascular accesses [11]. The length of the target lesion was 200 mm + −15 mm SD. The first-line strategy used in the endovascular group was balloon angioplasty; in all cases, nitinol self-expandable stents were used only for residual stenosis and flow-limiting dissections (Table 3). All the endovascular procedures were performed by a single vascular surgeon while the open bypasses were performed by experienced vascular surgeons. Technical failure occurred in 4.7% of cases in the endovascular group, compared to 0% in the open group (*p* = 0.047). In the endovascular group, failure occurred only in cases with completely occluded calcified lesions, regardless of their extension in length. The failures were characterised by failure to cross the lesion with the guidewire in three cases and failure to re-enter the true lumen after crossing it sub-intimally in the remaining two. These five limbs underwent femoro-popliteal bypass surgery, above the knee in three cases, using a heparin-bonded expanded polytetrafluoroethylene (ePTFE) graft, and below the knee in the remaining two cases, using the ipsilateral great saphenous vein. In the open group, the length of the target lesion was 205 mm + −18 mm SD. In the open group, all the bypasses were performed using a heparin-bonded ePTFE graft (GORE^®^ PROPATEN^®^ Vascular Graft, W. L. Gore & Associates, Inc., Newark, DE, USA). The inflow vessel was the common femoral artery, and the outflow vessel was the popliteal artery, above the knee in 41 limbs and below the knee in 39 (Table 3).

**Figure 2:**
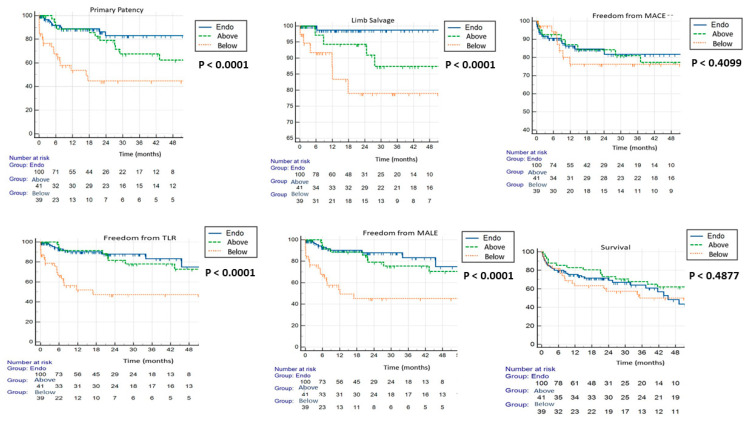
Kaplan–Meier curves for primary patency, limb salvage, freedom from TLR, freedom from MALE, freedom from MACE and survival rate. MACE, major adverse cardiovascular event; MALE, major adverse limb event; TLR, target lesion revascularisation.

Clinical outcomes. Compared to the open group, the endovascular group showed superior primary patency (*p* < 0.0001), cdTLR (*p* < 0.0001), MALE (*p* < 0.0001), and freedom from limb loss (*p* < 0.0001) at 12, 24 and 48 months. Further analysis of the data for the open above-the-knee subgroup showed that the aforementioned endpoints were similar between the two groups at 12 months. At 24 and 48 months, the outcomes favoured the endovascular group altogether (primary patency *p* = 0.4 and <0.01, freedom from cdTLR *p* = 0.08 and 0.89, freedom from limb loss *p* < 0.01 and <0.001, freedom from MALE *p* = 0.02 and 0.04, respectively). Freedom from MACE and survival rates were similar in both groups (Figure 2) throughout the duration of the follow-up. The procedural times and in-hospital stays were longer in the open group than those in the endovascular group (*p* < 0.0001 and *p* < 0.001, respectively). Post-procedural haemoglobin loss was more evident in the open group than in the endovascular group. More complications related to vascular access and groin incision, such as dehiscence, infection, lymphorrhoea, and haematoma, were observed in the open group than in the endovascular group (Table 4). In the percutaneous endovascular group, two groin haematomas were identified using an ultrasonographic examination after groin swelling occurred; the haematomas were managed conservatively by compression. One case of acute thrombosis occurred due to unsuccessful percutaneous closure device deployment, which needed surgical revision.

Cost of procedural materials. The cost of materials used for the procedures was 10-fold lower in the endovascular group than in the prosthetic bypass group. Heparin-bonded ePTFE grafts were used in all cases. In the open group, the cost of the materials used did not differ from one procedure to the next and ranged around EUR 2800 ± SD 200 per procedure. In contrast, in the endovascular group, expenditure depended on the type and number of devices, such as guidewires, balloons, and stents, that were used. For a standard procedure, expenditure fluctuated around EUR 243 ± SD 150 and increased if paclitaxel-coated devices (costing EUR 590–850) were used instead of bare metal stents (EUR 340), and with the number of stents delivered. Details are presented in Table 3; the manufacturers’ names are mentioned only for transparency, and not for publicity.

## 4. Discussion

Traditionally, complex atherosclerotic femoro-popliteal lesions have been treated using a femoro-popliteal bypass, with vein grafts being used as conduits, as they produce the best results based on long-term patency [5,12,13]. Several situations can lead to a prosthetic graft being chosen, including the unavailability of a suitable vein graft. In select above-the-knee cases, femoro-popliteal bypass is required, and most surgeons tend to retain the vein for more distal revascularisations. However, in many centres, the endovascular approach—which was once reserved for use with short TASC II A and B lesions and frail patients—is currently considered as an alternative to open surgery, even for complex lesions. The continuous technological advances being made in the endovascular field, which have introduced numerous devices dedicated to each stage of the procedure, have made this possible. The advances have led to a substantial improvement in outcomes based on technical success and long-term primary patency [14,15,16,17,18]. In this study, we aimed to evaluate whether using the endovascular approach for long complex lesions could offer any advantages over using the femoro-popliteal prosthetic bypasses approach, regardless of lesion features and anaesthesiological risks. The value this study holds is related to the homogeneity of both groups regarding treated lesions, region affected, clinical limb presentation, and ASA risk scores.

We found that open surgery enabled superior completion of the revascularisation of the index limb. In our experience, technical failure was mainly influenced by the calcification of the lesions. Calcium is considered a strong predictor of technical failure and loss of patency, and several models, such as the peripheral artery and Fanelli scoring systems, have been presented to predict outcomes [19,20]. Only complete vessel occlusion influenced the technical success of the procedures analysed in this study, not the extended length of the calcifications. We believe that these lesions are the most challenging to cross and, in most cases, the subintimal approach is the only strategy that can be used. However, the 4.7% technical failure rate in our study was significantly lower than the 20% technical failure rate for the endovascular procedures reported in the BASIL I trial, which compared the outcomes in the open and endovascular arms for complex femoro-popliteal lesions [13]. The data reported for the BASIL I study should be considered out-dated, as the technical success achieved with new-generation devices is approximately 99% [21]. The main technical issues in these cases are related to failure to cross the lesion. Moreover, in some cases, the lesion is crossed in a sub-intimal manner to find the distal re-entry point. To overcome these issues, guidewires for specific lesions and stages of the procedure have been developed and are available in every operating room and angiographic suite; several devices on the market have been proven safe and efficacious in terms of technical success [22].

Another issue is that of how devices such as balloons and stents are delivered once the lesion has been crossed with a guidewire; in some cases, even attempting to cross the lesion with a small-diameter balloon for pre-dilatation may fail. Generally, this type of failure occurs in heavily calcified lesions. In these cases, using atherectomy to remove the plaque, even partially, becomes useful to enable the delivery of the aforementioned devices [23,24]. These devices substantially increase procedural cost; however, they also increase the success rate of the endovascular procedure for complex lesions. The use of adjunctive procedures, such as atherectomy and provisional stenting, are required when dealing with complex femoro-popliteal lesions [25].

Another widely adopted aspect is that many centres offer endovascular treatment as a minimally invasive approach for frail patients, as observed in our study. In our cohort, patients treated endovascularly had significantly more co-morbidities and were older than the patients treated with open surgery. However, every choice of treatment should be justified by improved clinical and long-term outcomes based on primary patency, cdTLR, MALE, and survival. Angioplasty with only simple balloons is known to result in poor outcomes for long lesions. However, performing adjunctive procedures such as atherectomy or nitinol stenting as a primary or bailout strategy, and utilising drug-coated devices, substantially improves the clinical outcomes [18,26,27]. Drug-coated devices or provisional stents were not used in the BASIL I trial, which may explain the poor primary patency outcomes reported in the endovascular arm.

In the contemporary literature, the main comparisons reported involve endovascular and vein-bypass revascularisation of the limb. The BEST-CLI trial highlighted better outcomes for revascularisations performed with a single suitable vein conduit, and similar outcomes between endovascular and open revascularisations performed with an alternative conduit; however, the trial did not focus on a particular approach [5]. In contrast, in the BASIL II trial, endovascular procedures were reported to provide better performance than vein bypasses in the infrapopliteal arteries [28]. Few studies have directly compared endovascular and prosthetic graft revascularisations in long femoro-popliteal lesions and reported similar results for the endovascular approach and the use of an ePTFE conduit [29]. In contrast, in our series, using the open approach showed no advantages over using the endovascular approach, as the overall long-term outcomes were worse in the open group than those in the endovascular group. In the above-the-knee sub-group, the primary patency outcomes were similar between the two approaches for the first 24 months, and the endovascular arm showed more favourable outcomes thereafter. For these patients, using an endovascular approach as a first-line strategy would have provided benefits in terms of operating time, intra-operative blood loss, fast functional recovery, and early discharge. Moreover, in our study, the endovascular approach was beneficial in terms of vascular access management and complications rate. In this aspect, percutaneous procedures—especially those supported by ultrasound guidance during arterial puncturing and use of a percutaneous closure device to perform haemostasis—significantly favoured the endovascular choice, even in hostile groins [11,30,31,32].

Finally, in-hospital stay has become an important goal of public health management, which aims to reduce in-hospital management costs and favours patient turn-over [33,34]. The cost of the materials used in the open group must also be considered as they result in higher expenses being incurred than the standard endovascular procedure does. We did not use expensive devices, such as atherectomy, lithotripsy, intravascular ultrasound, or re-entry devices; this may explain the aforementioned cost differences found in this study. However, the costs incurred for patients in the open group were related to our use of heparin-bonded ePTFE grafts, which are expensive.

### Limitations of This Study

The main limitation of this study was its retrospective nature. The presence of a sub-group wherein vein grafts were used may have benefitted this study. However, applying specific criteria helped us to obtain two homogeneous groups for comparison, and the use of several endpoints contributed to the overall comprehensiveness of this study.

## 5. Conclusions

Endovascular procedures can be safely performed in patients at a high risk for surgery and deliver good outcomes at 2 and 4 years. Endovascular procedures have better long-term outcomes for many endpoints than prosthetic bypasses do; thus, endovascular procedures could be considered the first-line strategy for use in treating complex femoro-popliteal lesions. Revascularisation with a prosthetic bypass remains a superior procedure in term of technical success, even in cases where the endovascular approach has failed.

## Figures and Tables

**Table 1:** Demographic and co-morbidity-related information for the patients in the open and endovascular groups. COPD, chronic obstructive pulmonary disease; CAD—coronary artery disease, * History of ischaemic stroke.

Demographics Co-Morbidities	Open Group (n = 76)	Endovascular Group (n = 98)	*p*-Value
Mean age, years	74 ± 8 SD	79 ± 9 SD	-
Age ≥ 80 years	20 (26.3%)	40 (40.8%)	0.045
Age ≥ 90 years	0 (0%)	6 (6.1%)	0.028
Men	49 (64%)	59 (60%)	0.5648
Women	27 (36%)	39 (40%)	0.5648
Current smokers	22 (29%)	18 (18%)	0.0999
Systemic hypertension	72 (95%)	90 (92%)	0.4539
Dyslipidaemia	44 (58%)	62 (63%)	0.4714
Diabetes	34 (45%)	47 (48%)	0.6725
Chronic renal disease	19 (25%)	27 (28%)	0.7050
CRD dialysis	8 (11%)	9 (9%)	0.7673
Coronary artery disease	35 (46%)	66 (67%)	0.0047
COPD	13 (17%)	15 (15%)	0.7487
Stroke *	7 (9%)	22 (22%)	0.0201

**Table 2:** The quality of below-the-knee run-off vessels for the two groups. BTK, below-the-knee.

Run-Off	Open Group (n = 80 Limbs)	Endovascular Group (n = 105 Limbs)	*p* Value
Number of BTK vessels	Number of limbs	Number of limbs	
0	0	11	0.002
1	30	40	0.462
2	22	44	0.042
3	28	10	0.001

**Table 3:** The technical features of the open and endovascular groups regarding the devices, and types of grafts and vascular access chosen. POBA, plain old balloon, (* semi compliant balloon, Medtronic Plymouth, MN, USA), DCB, drug-coated balloon (^#^ paclitaxel balloon, CR Bard Becton Dickinson Franklin Lakes, NJ, USA); pBMS, provisional bare-metal stent (^&^ Cook Medical Medical, Bloomington, IN, USA); pDES, provisional drug-eluting stent (paclitaxel); SFA, superficial femoral artery.

Open Group	Number of Limbs (%) (n = 80)
Above the knee	41 (51.2%)
Below the knee	39 (48.7%)
**Graft material**	
Heparin-bonded ePTFE	80 (100%)
**Graft diameter**	
6 mm	38 (47.5%)
7 mm	36 (45%)
8 mm	6 (7.5%)
**Endovascular group**	**Number of limbs (%)** **(n = 105)**
**Vascular access**	
Cut down	53 (50.4%)
Percutaneous	52 (49.5%)
Ipsilateral	12 (11.4%)
Contralateral	40 (38%)
**Haemostasis**	
Percutaneous closure device (AngioSeal Vip 6 Fr)	52 (49.5%)
Surgical suture	53 (50.4%)
**Target lesion**	
SFA	71 (67.6%)
SFA and popliteal artery	34 (32.3%)
**Endovascular devices**	
POBA (Evercross) *	105 (100%)
DCB (Lutonix) ^#^	4 (3.8%)
pBMS (Zilver Flex) ^&^	24 (22.8%)
pDES (Zilver PTX) ^&^	5 (48.7%)

**Table 4:** Characteristics of the surgeries showing the anaesthesiologic approach, adjunctive procedures, intervention course, complications at the groin access, and post-operative medications in the two groups. * All complications occurred at the groin. # Conditions overlapped at the same surgical incision and occurred within 30 days. CFA, common femoral artery; DAPT, dual antiplatelet therapy.

Anaesthesiologic Management	Open Group (n = 80)	Endovascular Group (n = 105)	*p*-Value
General	71 (87.5%)	41 (39%)	<0.00001
Local	0 (0%)	37 (35.2%)	<0.00001
Local + systemic sedation	0 (0%)	17 (16.1%)	0.0001
Epidural analgesia	9 (2.5%)	5 (4.7%)	0.434
**Adjunctive procedures**			
CFA endarterectomy	17 (21.2%)	1 (0.9%)	<0.001
CFA and PFA endarterectomy	4 (5%)	0 (0%)	0.02
CFA angioplasty	0 (0%)	22 (20.9%)	<0.001
Popliteal endarterectomy	8 (10%)	0 (0%)	0.001
Minor amputations	14 (17.5%)	13 (12.3%)	0.328
**Technical success**	0 (0%)	5 (4.7%)	0.047
Overall intervention duration, minutes *	273 ± 96	146 ± 89	<0.0001
Duration of the intervention, minutes *	**Open group** **(n = 80)**	**Endovascular percutaneous group** **(n = 52)**	
	273 ± 96	78 ± 37	<0.00001
**Haemoglobin loss ≥ 3 g/dL**	13 (16.25%)	6 (5.7%)	0.019
**Overall vascular access #**	9 (11.2%)	3 (2.8%)	0.021
Dehiscence	6	0	
Infection	4	0	
Lymphorrhoea	6	0	
Haematoma	1	3	
**Post-intervention therapy**			
DAPT 3 months	52 (65%)	80 (76.1%)	-
Anti-coagulant	13 (16.2%)	0 (0%)	-
Anti-coagulant plus anti-platelet	15 (18.7%)	25 (23.8%)	-

## Data Availability

Not applicable.
